# Impulse Control Behaviors in Parkinson's Disease: Drugs or Disease? Contribution From Imaging Studies

**DOI:** 10.3389/fneur.2018.00893

**Published:** 2018-10-25

**Authors:** Rosa De Micco, Antonio Russo, Gioacchino Tedeschi, Alessandro Tessitore

**Affiliations:** ^1^Department of Medical, Surgical, Neurological, Metabolic and Aging Sciences, University of Campania “Luigi Vanvitelli, ” Naples, Italy; ^2^MRI Research Center SUN-FISM, University of Campania “Luigi Vanvitelli, ” Naples, Italy

**Keywords:** Parkinson's disease, impulse control behaviors, MRI, PET, reward system

## Abstract

Impulse control behaviors (ICB) are recognized as non-motor complications of dopaminergic medications in patients with Parkinson's disease (PD). Compelling evidence suggests that ICB are not merely due to the PD-related pathology itself. Several risk factors have been identified, either demographic, clinical, genetic or neuropsychological. Neuroimaging studies have yielded controversial results regarding ICB correlates in PD and still it is not clear whether they can be triggered by the PD biology or the dopaminergic treatment stimulation. We provided an overview of the imaging studies that offered the most relevant insights into the debate about the role of drugs and disease in ICB pathophysiology. Understanding neural correlates and potential predisposing factors of these severe neuropsychiatric symptoms will be crucial to guide clinical practice and to foster preventive strategies.

## Introduction

Impulse control behaviors (ICB) are neuropsychiatric symptoms characterized by impulsive acts, which are performed compulsively and are potentially detrimental to the person itself or others, severely affecting subjects' quality of life (1). ICB are mainly recognized as side-effects of treatment with dopaminergic medications in patients with Parkinson's disease (PD) (2). Compelling evidence suggests that ICB are not merely due to the disease-related pathology itself (3). The lifetime prevalence of ICB in PD patients ranges between 6–9% and increases to 14% in patients taking dopamine-replacement therapy such as dopamine agonists (DAA) or levodopa (2, 3). The risk to develop ICB increases of 2- to 3.5-fold when patients are exposed DAA (2). The prevalence did not differ between the two commonly prescribed oral short-acting DAA, pramipexole and ropinirole (17.7 vs. 15.5%) with a relatively low rate of ICB with long-acting and transdermal DAA (6.6% pramipexole prolonged release and 4.9% for rotigotine) (2, 4). However, not all PD patients develop ICB under dopaminergic treatment. Several risk factors have been proposed, either clinical (i.e., younger age at PD onset, male sex, depression) (3) or genetic (i.e., polymorphisms in dopaminergic, glutamatergic and serotoninergic and opioid receptors) (5, 6).

Interestingly, ICB also occur in patients with restless legs syndrome (7, 8) or prolactinoma (9) under DAA, and this may support the role of the drugs in triggering these neuropsychiatric symptoms.

Modern neuroimaging techniques have been widely tested to support PD diagnosis (i.e., positron emission tomography, PET and single-photon emission computed tomography, SPECT) as well as to provide further insights into motor and non-motor symptoms pathophysiology, complications and treatment-related effects (i.e., PET and magnetic resonance imaging, MRI) (10).

PET and SPECT studies have been extensively applied to analyze neural correlates underpinning ICB in PD (11–14). By means of pre- and post-synaptic tracers, these studies provided crucial insights about the nigrostriatal functioning characterizing ICB patients. Recently, highly selective D2/D3 tracers have been also implemented, allowing to detect the presence of widespread extra-striatal changes related to ICB.

Structural MRI changes have been also observed in PD patients with ICB. Gray matter atrophy as well as corticometric changes across several brain areas involved in behavioral modulation (i.e., orbitofrontal and anterior cingulate cortices) are the most frequent findings related to the ICB presence and severity in PD (15, 16). However, there is also evidence of no morphometric changes (17).

Functional MRI studies (18–21) were performed in resting condition as well as during reward tasks in ICB patients, and were used to shed light on specific reward processing abnormalities. Overall, these studies have consistently demonstrated a dysfunction within and between dopaminergic neural circuitries involving crucial subcortical hubs (i.e., ventral striatum, VS, amygdala) and limbic-cognitive cortical areas (i.e., anterior cingulate and frontal cortices). Interestingly, the most relevant brain areas in the pathogenesis of ICB are involved in the so-called neurocognitive networks, namely the default-mode (DMN), the salience (SN) and the central-executive (CEN) networks.

The DMN encompasses mainly precuneus and posterior cingulate, bilateral inferior-lateral-parietal and ventromedial frontal cortices. It is involved in cognitive processing and mind-wandering and becomes deactivated during specific goal-directed behaviors. The CEN is involved in executive control and decision-making and operates through medio-frontal areas, including anterior cingulate and para-cingulate cortices. The SN is a limbic-paralimbic network that plays an important role in orienting attention toward salient stimuli and facilitating goal-directed behaviors, reward processing and interoceptive awareness (22). It encompasses manly the dorsal anterior cingulate cortex and the bilateral VS. The dynamic interplay between these networks is critical to allow an individual to be behaviorally and cognitively efficient (23), and this highlights their potential relevance in ICB pathophysiology.

Overall, most imaging studies applied cross-sectional designs, yielding controversial results. Thus, it is not possible to rule out whether these findings reflect the effect of chronic dopaminergic treatment or represent a neural pattern predisposing to ICB (24).

Here, we aim to review the most relevant imaging studies that provide a contribution to the debate about the role of drugs and disease in the pathogenesis of ICB in PD.

## Search strategy

Articles published on PubMed until June 2018 were checked for the purpose of this review. “Parkinson's disease” were cross-referenced with “impulse control disorders” and synonymies and “magnetic resonance imaging,” “positron emission tomography,” “impulsive compulsive disorders,” “reward system,” “dopamine agonists,” “levodopa.” Two independent observers (RDM and AR) evaluated the results (*n* = 984), excluding duplicates and articles judged irrelevant by title and abstract screening. The same raters performed the quality check of selected studies and the most relevant ones for the topic were finally included in the review (Table 1).

**Table 1:** Summary of the methods and results from the studies included in the review.

**References**	**Imaging methods**	**Subjects**	**ICB screening**	**Main findings**
Ray et al. (25)	[11C]FLB-457 PET	7 PD patients with PG vs. 7 PD patients without PG	G-SAS	Decreased midbrain D2 and D3 autoreceptor sensitivity during a gambling task in patients with PD and PG compared with those without
Steeves et al. (11)	[11C]raclopride PET during gambling task	7 PD patients with PG vs. 7 PD patients without PG	Clinical interview, DSM-IV-TR	Decreased binding potential in the VS in PG patients than control patients at rest and during gambling task
O'Sullivan et al. (26)	[11C]raclopride PET during gambling task, before and after a levodopa challenge	11 PD patients with ICB vs. 7 PD patients without ICB	Semi-structured interview	Decreased binding potential in the VS in PD-ICB patients compared to control patients following reward-related cue exposure
Stark et al. (27)	[18F]fallypride PET	17 PD patients with ICB vs. 18 PD patients without ICB	Clinical interview and QUIP-RS	Lower binding potential within the VS and putamen in ICB patients compared with those without ICB
Cilia et al. (28)	[123I]FP-CIT SPECT	8 PD patients with PG vs. 21 PD patients without PG vs. 14 healthy controls	Clinical interview, DSM-IV-TR	Lower DAT binding in PD patients with PG compared to PD patients without PG
Voon et al. (13)	[123I]FP-CIT SPECT	15 PD patients with ICB vs. 15 PD patients without ICB	Clinical interview, DSM-IV-TR	Lower DAT binding in PD patients with ICB compared to PD patients without ICB
Politis et al. (19)	fMRI during sexual-cues exposure before and after levodopa challenge	12 PD patients with HS vs. 12 PD patients without HS	Clinical interview, DSM-IV-TR	Higher activity within the salience network in PD patients with HS compared to PD patients without HS during sexual cues, enhanced by levodopa administration
Tessitore et al. (20)	Resting-state fMRI	15 PD patients with ICB vs. 15 PD patients without ICB and 24 healthy controls	Clinical interview, MIDI	Increased connectivity within the salience and default-mode networks, and decreased connectivity within the central executive network in ICB-PD patients compared to those without
Tessitore et al. (21)	Resting-state fMRI	15 drug-naïve PD patients which developed ICB after treatment initiation vs. 15 drug-naïve PD patients who did not	Clinical interview, QUIP-RS	Baseline decreased connectivity in the default-mode and central executive networks and increased connectivity in the salience network in PD patients with ICB at follow-up compared with those without
Vriend et al. (14)	[123I]FP-CIT SPECT	11 drug-naïve PD patients which developed ICB after treatment initiation vs. 20 drug-naïve PD patients who did not	Clinical interview	Baseline lower DAT binding in PD patients with ICB at follow-up compared with those without
Voon et al. (18)	fMRI during reward task before and after DAA intake	14 PD patients with ICB vs. 14 PD patients without ICB	Clinical interview, DSM-IV-TR	After DAA treatment, PD-ICB patients present enhanced sensitivity to risk compared to PD patients without ICB
van Eimeren et al. (29)	[H152O] PET before and after DAA intake	7 PD patients with PG vs. 7 PD patients without PG	Clinical interview	DAA intake reduces cerebral blood flow in cortical areas involved in impulse control and behavioral inhibition
van der Vegt et al. (30)	fMRI during reward task	13 drug-naive PD patients vs. 12 healthy controls	Not applicable	Decreased neural response to reward outcomes within mesolimbic and mesocortical regions in drug-naïve PD patients compared to healthy controls
Thaler et al. (31)	fMRI during reward task	36 non-manifesting carriers of LRRK2 mutation vs. 32 non-manifesting non-carriers	Not applicable	Reduced activations upon risky anticipation and punishment in the VS and insula and higher activation upon safe anticipation in the insula in non-manifesting carriers

*ICB, impulse control behaviors; fMRI, functional MRI; PET, positron emission tomography; SPECT, single-photon emission computed tomography; PG, pathological gambling; HS, hypersexuality; VS, ventral striatum; G-SAS, gambling symptom assessment scale; QUIP-RS, Questionnaire for Impulsive-Compulsive Disorders in Parkinson's Disease-Rating Scale; DSM-IV-TR, Diagnostic and Statistical Manual of Mental Disorders Text Revision criteria; MIDI, Minnesota Impulsive Disorders Interview; DAA, dopamine-agonist*.

## Neuroimaging studies to analyze dopaminergic signaling in PD patients with ICB

The role of dopaminergic signaling in ICB development is suggested by both PD pathophysiology and DAA targeting. The most prescribed DAA are highly selective on D3 receptors, which are mainly located in the mesolimbic circuit and are thought to be involved in the reward processing (32). Interestingly, animal studies showed that nigrostriatal degeneration itself may result in increasing rewarding properties of D2 and D3 agonists in the mesolimbic pathway (33). Polymorphisms in D2 as well as D1-like receptors genes, potentially leading to abnormal neurotransmitters functioning, have been linked to increased ICB susceptibility in PD (6). On the other hand, chronic dopaminergic treatment may induce long-term abnormalities in the phasic and tonic activity of dopaminergic neurons, potentially leading to changes in post and pre-synaptic receptors density and properties (34, 35). In preclinical studies, these changes have been linked to reward anticipation and risk-taking behaviors [see for a review (24)]. Taken together these findings suggest that both disease and drugs seem to be synergistically involved in triggering ICB symptoms.

Neuroimaging studies are in line with this evidence. Indeed, in a small PET study using [11C]FLB-457, a radiotracer with high affinity for extra-striatal receptors, decreased midbrain D2 and D3 autoreceptor sensitivity have been shown during a gambling task in patients with PD and gambling compared with those without (25). This may reflect enhanced striatal dopamine release in PD patients with ICB when exposed to reward stimuli. Two PET studies (11, 26) found that PD-ICB patients present decreased [11C]raclopride binding potential in the VS during reward cues exposure compared to PD patients without ICB. As [11C]raclopride is highly selective for post-synaptic D2 receptors, a reduced binding may suggest again the presence of a “hyperdopaminergic state” in the VS of patients with PD-ICB. This effect was observed in “off” condition, as well as after a levodopa challenge (26). Interestingly, no binding change was determined by levodopa intake upon neutral cues (26). Recently, more selective tracers have been implemented, such as [18F]fallypride, which is a high affinity D2-like receptors ligand that can measure D2/D3 binding potential throughout the meso-cortico-limbic network. This tracer was used in a cohort of PD patients with ICB compared with those without, confirming that the presence of a reduced binding potential within the VS and putamen may be a marker of increased dopaminergic levels (27). Moreover, this study showed that the integrity of the dopaminergic projections emerging from the midbrain differentiates PD patients with ICB from those without, and increases along with severity of symptoms (27). This finding is in line with the hypothesis that ICB may result from the imbalanced involvement of the more affected dorsal and the less affected VS in the early stages of PD. Thus, while dopaminergic treatment partially restores the normal functioning within the dorsal striatum (improving motor symptoms), the dopaminergic treatment may “overdose” the VS, potentially triggering affective disturbances and ICB (36, 37).

Other neuroimaging approaches have confirmed the presence of dopaminergic signaling abnormalities in patients with PD and ICB. Indeed, reduced striatal dopamine transporter (DAT) density has been reliably reported in PD-ICB patients compared to PD patients without ICB (13, 28). This is of interest, as the DAT binding may decrease following either mesolimbic projections neurodegeneration or increased dopaminergic synaptic firing.

In a functional MRI (fMRI) study, patients with PD and hypersexuality exposed to sexual cues had higher activity within the SN compared to patients with PD without ICB (19). Moreover, this study showed that subjective sexual desire was enhanced by levodopa administration (19). A similar pattern of increased SN connectivity has been also shown at rest in PD patients with ICB compared to those without (20). Functional connectivity abnormalities were also found to be correlated to ICB severity (20).

In summary, different neuroimaging techniques have been used to analyze the integrity of striatal and extra-striatal dopaminergic pathways in PD patients with and without ICB. The presence of a specific “hyperdopaminergic” state in the brain of patients experiencing ICB have been consistently highlighted. An important limitation is that these studies have mainly enrolled PD patients with a long history of PD as well as ICB, which may both influence the reward and impulse-control pathways themselves. Indeed, after ICB emergence, progressive neuroplasticity processes involving mainly dopaminergic circuitries may occur, eventually leading to consolidation of pathological habits (38). Thus, even though with caution, these studies corroborate the idea that PD-related pathology and dopaminergic treatment may synergistically act on the risk to develop ICB in PD patients.

## Neuroimaging studies to analyze reward processing in PD patients with ICB

Dopaminergic medications can influence rewarding processing, by enhancing learning from positive feedback and impairing learning from negative feedback (39, 40). Moreover, these drugs has been link to increased impulsivity (24).

Reward processing changes after dopaminergic drugs administration has been studied in healthy subjects as well as in patients with restless legs syndrome in order to describe pharmacological effects not biased from neurodegenerative pathology. Pessiglione et al. (41) performed a fMRI study to assess the effects of either levodopa (100 mg) or an antagonist of dopamine receptors (1 mg of haloperidol) on both brain activity and behavioral choice in healthy subjects. They found that during instrumental learning, levodopa increases while haloperidol reduces dopaminergic functioning in the VS along with the magnitude of reward prediction error. Accordingly, compared to subjects treated with haloperidol, subjects treated with levodopa showed greater propensity to choose the most rewarding action, supporting the hypothesis that dopamine-dependent modulation of striatal activity can account for how the healthy brain uses prediction errors to modulate future decisions (41). Another crucial component of the reward processing is the temporal impulsivity, which is the preference for smaller but sooner over larger but later rewards (42). This phenomenon is related to an excessive discounting of future rewards and has been observed in patients with drugs addiction (43). This function was tested in a cohort of young healthy subjects by means of a task-related and pharmacological fMRI paradigm (44). The study revealed that levodopa increases preference for more immediate rewards, likely increasing impulsivity in healthy brains. This result parallels with a corresponding increased neural representation in the striatum, further supporting the idea that a hyperfunctioning in the dopamine system is related to abnormal decision-making.

Along with levodopa, the effect of DAA treatment on the reward processing was tested in both healthy and non-healthy subjects as well. A double-blind study compared results from a probabilistic reward task performed after either a single low dose of pramipexole (0.5 mg) or placebo (45), revealing that DAA may affect the acquisition of reward-related behaviors (45). A similar effect was found also in a cohort of subjects with restless legs syndrome without any history of pathological gambling (46). In this study, fMRI scans were obtained during a gambling game task, once whilst subjects were taking their regular medication (i.e., low dose DAA) and after a washout period. Upon expectation of rewards, significant VS activation was detected only when subjects were taking DAA, but not when they were in the washout period. Contrariwise, upon omission of rewards, the observed VS signal under DAA were significantly different from what revealed during the washout (46).

These results parallel with several evidence coming from PD patients with ICB. A task-related and pharmacological fMRI study performed before and after DAA treatment, showed that PD-ICB patients under DAA present enhanced sensitivity to risk compared to PD patients without ICB in the same experimental condition (18). DAA intake has been also shown to reduce cerebral blood flow in cortical areas involved in impulse control and behavioral inhibition (29).

However, it should be noted that PD results from the degeneration of dopaminergic projections involved in the reward processing itself. By contrast, dysfunctions within the reward system are difficult to study in PD as most patients are treated with dopaminergic drugs. In this context, a fMRI task-related paradigm was used in a small group of drug-naïve PD patients performing a simple two-choice gambling task (30). In this study, PD patients compared to healthy controls showed decreased neural response to reward outcomes within several mesolimbic and mesocortical nodes, such as the ventral putamen, ventral tegmental area, thalamus and hippocampus. In this framework, reward processing abnormalities were also found in subjects at high risk for future development of PD, such as a cohort of non-manifesting carriers of the G2019S mutation in the LRRK2 gene (31). Indeed, this event-related fMRI study showed differences between non-manifesting carriers and non-carriers when comparing activations in key reward brain areas upon safe and risky anticipation and punishing outcomes. Thus, several nodes of the meso-cortico-limbic reward system are already compromised in the early (and also preclinical) stages of the disease as they are also direct targets of PD-related neurodegeneration.

In summary, even in the absence of manifest ICB symptoms as well as PD pathology, chronic dopaminergic medication was shown to severely impair the reward processing. Although limited, neuroimaging evidence of altered reward-processing in PD patients even in the absence of DAA treatment have been provided.

## Neuroimaging studies to predate ICB development in PD patients

To date, only a few studies have been designed to find potential neuroimaging biomarkers able to predict future development of ICB in PD. This is crucial, as previous studies did not allow to disentangle the complex interplay between drugs and disease in ICB pathophysiology. Vriend et al. (14) performed a retrospective analysis of DAT imaging data acquired in a cohort of drug-naïve PD patients that developed ICB symptoms after dopaminergic treatment initiation. They found that the presence of reduced DAT availability in the VS at baseline is able to predate ICB development after treatment initiation. Dopamine reuptake via striatal DAT is the most important mechanism acting to remove dopamine from the synapse. Thus, PD patients with lower DAT availability could have increased striatal dopamine levels (14, 28) even at the time of the diagnosis. This important finding corroborates the hypothesis that PD patients with higher risk to develop ICB may present at baseline a relatively preserved striato-cortical functioning. As we mentioned above, increased dopaminergic signaling in the VS can interfere with the processing of negative feedback during reward-based learning. Neurobehavioral studies (47, 48) have shown that the high dopaminergic firing occurring upon reward cues is able to reinforce hippocampal inputs and inhibits prefrontal connections on the VS. In the absence of feedback top-down processes, this divergent effect may impair the ability to shift behavioral focus when cues salience change, potentially looping the reward system. A similar condition may occur in PD patients with a “hyperdopaminergic” state in the VS (i.e., patients at higher risk to develop ICB) and then exposed to the dopamine-mimetic treatment (49), leading to impulsive-compulsive behaviors. However, further investigations are warranted to clarify which predisposing factors, potentially genetic (5, 6), may determine this trend toward increased dopaminergic response. In this framework, different polymorphisms in several neurotransmitters receptors genes, potentially leading to high dopaminergic striatal levels, have been linked to increased ICB susceptibility in PD (6).

More recently, resting-state fMRI was used to analyze the intrinsic functional connectivity within and between the major neurocognitive networks in a cohort of drug-naïve PD patients that developed ICB (ICB+) after treatment initiation compared with PD patients who did not (ICB–) (21). In physiological condition, the SN modulates the inter-network connectivity between the CEN and the DMN, resulting in a functional anti-correlation between these two networks (23, 50). This dynamic balance is crucial, as it is thought to drive an efficient behavioral and cognitive outcome (23). When comparing ICB+ and ICB- patients before treatment initiation, an increased resting-state connectivity within the SN was found in ICB+ patients (21). This is of interest, as the SN encompasses cortical and subcortical nodes that are affected by PD-related pathology itself, such as the VS (51, 52). The presence of an increased connectivity within this network may again rely on pre-existing abnormal dopaminergic signaling even at the disease onset, and may also explain the development of such behavioral complications when patients are exposed to dopaminergic medication. Interestingly, the study also revealed that the anti-correlation between DMN and CEN is lost at the time of diagnosis in ICB+ patients and this inverse pattern showed a positive correlation with the time to ICB onset (i.e., the less the anti-correlation between DMN and CEN the earlier is the emergence of ICB). Notably, no differences have been shown between ICB+ and ICB- patients in terms of total levodopa equivalent daily dose (including DAA) at the time of ICB development (21). Thus, these connectivity changes may represent a potential biomarker to predict emergence of ICB symptoms before starting any dopaminergic drugs.

In summary, longitudinal neuroimaging studies on premorbid ICB population are limited. However, they support the hypothesis that a pre-existing vulnerability to ICB development may be present in a specific subset of PD patients, likely related to PD-pathology, involving both dopaminergic signaling and reward processing, which are in turn affected by dopaminergic medications.

## Conclusions

The relationship between PD pathology, DAA treatment and ICB development is complex. Neuroimaging studies have provided crucial insights to support the presence of increased dopaminergic firing in response to reward stimuli in the cortico-striato-cortical pathway in PD patients more prone to develop ICB. Dopaminergic treatment exposure may overdrive this pathway and also induce further dopamine receptors changes, leading to the development of such behavioral disturbances. Future multimodal imaging studies able to look at several aspects of the dopaminergic cortical and subcortical signaling, as well as prospective longitudinal designs, will allow to disentangle how drugs and disease may interplay to trigger these relevant neuropsychiatric symptoms. Understanding neural correlates and potential predisposing factors of these severe behavioral symptoms will be crucial to guide clinical practice and to foster preventive strategies.

## Author contributions

RD study concept and design, acquisition of data, drafting the article. AT drafting the article and revising it critically for intellectual content. AR critically revision of manuscript for intellectual content. GT critically revision of manuscript for intellectual content.

### Conflict of interest statement

The authors declare that the research was conducted in the absence of any commercial or financial relationships that could be construed as a potential conflict of interest.
